# Performance and standards for the process of head and neck cancer care: South and West audit of head and neck cancer 1996–1997 (SWAHN I)

**DOI:** 10.1054/bjoc.2000.1302

**Published:** 2000-07-24

**Authors:** M A Birchall, D Bailey, A Lennon, on behalf of the South and West Regional Cancer Organisation Tumour Panel for Head and Neck Cancer

**Affiliations:** University Department of Otolaryngology Head and Neck Surgery, Southmead Hospital, Bristol, BS10 5ND, UK; Cancer Intelligence Unit, Highcroft, Romsey Road, Winchester, SO22 5DH, UK

**Keywords:** performance indicators, standards, process, audit, head and neck cancer

## Abstract

Evidence suggests wide variation in cancer care between different hospitals in the UK. To establish bench-marking data, we designed a prospective, 1 year regional study comparing key performance measures with established standards for the 28 hospital Trusts in the South and West of England involved in head and neck cancer care. 566 sequential patients with a newly-diagnosed head and neck cancer were included. Numbers referred and treated per hospital Trust were 1–58 and 1–65 respectively. 59% of patients received a pretreatment chest X-ray (standard 95%). 45% of patients were seen in a multidisciplinary clinic pretreatment (standard 95%), and this was proportional to the frequency of clinics held (*P*< 0.0001). Median number of cases treated per surgeon was 4 (1–26), and by radiotherapist was 10 (1–51). Times between parts of the process of oral cancer care were closer to the standards than those for laryngeal cancer. Two patients were entered into a clinical trial. One had a quality-of-life score. Thus, in 1996–1997, in the South and West of England, there were major discrepancies between actual performance and established standards in many fundamental aspects of head and neck cancer care. Re-audit is essential to determine if the implementation of the Calman–Hine report has resulted in improvements. © 2000 Cancer Research Campaign


*British Journal of Cancer* (2000) **83**(4), 421–425
© 2000 Cancer Research Campaign
doi: 10.1054/ bjoc.2000.1302, available online at http://www.idealibrary.com on IDEAL®

### Performance and standards for the process of head and neck cancer care: South and West audit of head and neck cancer 1996–1997 (SWAHN I)

**MA Birchall[1], D Bailey[2] and A Lennon[2] on behalf of the South and West Regional Cancer Organisation Tumour Panel for Head and Neck Cancer**

[1]University Department of Otolaryngology, Head and Neck Surgery, Southmead Hospital, Bristol, BS10 5ND, UK;[2] Cancer Intelligence Unit, Highcroft, Romsey Road, Winchester SO22 5DH, UK

**Summary** Evidence suggests wide variation in cancer care between different hospitals in the UK. To establish bench-marking data, we designed a prospective, 1 year regional study comparing key performance measures with established standards for the 28 hospital Trusts in the South and West of England involved in head and neck cancer care. 566 sequential patients with a newly-diagnosed head and neck cancer were included. Numbers referred and treated per hospital Trust were 1–58 and 1–65 respectively. 59% of patients received a pretreatment chest X-ray (standard 95%). 45% of patients were seen in a multidisciplinary clinic pretreatment (standard 95%), and this was proportional to the frequency of clinics held ($P < 0.0001$). Median number of cases treated per surgeon was 4 (1–26), and by radiotherapist was 10 (1–51). Times between parts of the process of oral cancer care were closer to the standards than those for laryngeal cancer. Two patients were entered into a clinical trial. One had a quality-of-life score. Thus, in 1996–1997, in the South and West of England, there were major discrepancies between actual performance and established standards in many fundamental aspects of head and neck cancer care. Re-audit is essential to determine if the implementation of the Calman–Hine report has resulted in improvements. © 2000 Cancer Research Campaign

**Keywords:** performance indicators; standards; process; audit; head and neck cancer

Head and neck cancer presents the greatest impairment to quality of life of any malignancy and its management is highly resource-intensive. Despite this, retrospective evidence suggests that the organization of care for these patients in the UK is significantly flawed. A recent questionnaire study showed great disparity in practice between individual clinicians (Edwards et al, 1999), while a more recent retrospective study by the same group showed that 40% of patients in three UK regions received non-standard treatment (Edwards and Johnson, 1999). We used a formal nominal group method to establish standards for the process of care (Birchall, 1997; 1998). These standards have since been included in a consensus document agreed by the members of the British Association of Otorhinolaryngologists, Head and Neck Surgeons, which gives guidance for the management of patients with head and neck cancer (Wilson, 1998b; Wilson 1998c; Wilson, 2000). We then tested standards for the first part of the process of care by 1-year prospective regional audit. The aim of the study was to obtain an accurate picture of performance across a wide range of hospitals, to obtain bench-marking data and to allow analysis of the 'patient journey' through from diagnosis to completion of care for head and neck cancers.

### METHODS

### Patients

To prevent patient selection bias, the audit was population-based. All residents of the South and West region (population 6.5 million) diagnosed with a primary head and neck cancer in the period 1 December 1996 to 30 November 1997 were included. Cancers of skin, thyroid and lip were excluded, as were histologically proven cases of melanoma and lymphoma. Cases were identified by monthly downloads of pathology reports or by clinician reporting using standard proformas developed and piloted by the Tumour Panel. Staging used the UICC TNM system (UICC, 1997).

*Received 29 June 1999*
*Revised 13 March 2000*
*Accepted 13 April 2000*

*Correspondence to:* MA Birchall

### Outcome measures

This study examined that part of the process before treatment. Primary outcome measures were numbers of cases presenting and treated by individual clinicians and Trusts, proportions of tumours staged and proportions of patients receiving a chest X-ray and being seen in a multidisciplinary clinic pretreatment. Secondary outcome measures were times between activities in the process of care, proportions of patients with advanced (T3/T4 stage) tumours receiving computerized tomographic (CT) or magnetic resonance imaging (MRI) scans, numbers of patients invited/recruited into clinical trials and numbers completing quality-of-life measurements.

### Data protection and assurance

Data was protected by a strict Security and Confidentiality Policy conforming to current conventions (Department of Health, 1996). Internal audit and peer-review methods were used to ensure accuracy and validity of information, supplemented by computerized validation checks. Comparison with the Cancer Register was used to ensure all cases were identified. A final quality-check was achieved by sampling notes corresponding to returned forms in three randomly-chosen centres.

### RESULTS

Forms were received for 566 cases. A further 61 possible cases were identified by pathology reports, but found to be outside the audit on examination of hospital notes. Completeness of information exceeded 80% in most categories. However, performance status recording was low (23%). 359 (64%) of patients were male. 86% of patients with glottic cancer were male, while oral cavity cancers split 56% male: 44% female. Females tended to present later. The age group 45–64 years tended to contain more advanced stage tumours than those aged over 65 years. Total numbers by site were: larynx 179; oral cavity 170; pharynx 119; salivary 50; other sites 48.

Numbers of patients referred and treated per Hospital Trust were (median and range) 21 (1–58) and 9 (1–65) respectively (Table 1). Three hospital Trusts (11%) treated more than 50 new patients, while 16 (57%) treated less than 20. The number of Trusts listed exceeds those participating in the audit since a few patients were referred for treatment to Trusts outside the region (e.g. London). In addition, six patients (1%) were treated in private hospitals and these have been grouped together.

The mean proportion of patients staged (standard 100%) was 88% (larynx), 88% (oral cavity) and 74% (other), with overall mean being 83%. Overall, 59% of patients received a pretreatment chest X-ray (standard 95%, Table 2). For advanced (T3/T4) tumours, the mean percentage receiving MRI or CT-scan was: larynx 55% (range 0–100, standard 90%); oral cavity 57% (range 0–100, standard 90%); ear/nose/sinus 44% (range 0–100, standard 100%) (Table 2).

45% of patients were seen in a multidisciplinary clinic pretreatment (range 15–88%; standard 95%), and this was proportional to the frequency of clinics held ($\chi^2_3 = 17.4$; $P = 0.00017$) (Figure 1). The median number of cases treated per surgeon was four (range 1–26) (Figure 2), and by radiotherapist was 10 (range 1–51) (Figure 3). For surgical consultants, 85 (90%) treated less than 20 new cases per annum, while the corresponding figure for radiotherapists and oncologists was 14 (67%) seeing less than 20 new cases per annum.

Times between parts of the process of oral cancer care were closer to the standards than those for laryngeal cancer (Table 3). Only two patients (0.4%) were entered into a clinical trial. One had a quality-of-life score (standard 100%).

**Table 1** Numbers of new patients presenting with head and neck cancer to and treated by hospital Trust. The differences in numbers between the two columns represents those patients who were either transferred to another hospital for treatment, elected for no treatment or died prior to treatment

| Hospital Trust (code number) | Number referred | Number treated |
|---|---|---|
| 1 | 38 | 38 |
| 2 | – | 2 |
| 3 | 36 | 39 |
| 4 | 1 | – |
| 5 | 5 | 4 |
| 6 | – | 1 |
| 7 | 27 | 27 |
| 8 | 6 | 1 |
| 9 | 1 | 1 |
| 10 | 7 | 3 |
| 11 | – | 1 |
| 12 | 10 | 9 |
| 13 | 3 | 3 |
| 14 | – | 1 |
| 15 | 48 | 49 |
| 16 | 32 | 34 |
| 17 | 58 | 65 |
| 18 | 32 | 31 |
| 19 | 51 | 56 |
| 20 | – | 1 |
| 21 | 14 | 14 |
| 22 | 28 | 27 |
| 23 | 47 | 59 |
| 24 | 29 | 30 |
| 25 | 3 | 2 |
| 26 | 15 | 12 |
| 27 | 27 | 34 |
| 28 | 27 | 23 |
| 29 | 2 | 1 |
| 30 | 12 | 8 |
| 31 | 7 | 6 |
| Private | – | 6 |
| Refused/none | – | 6 |

**Table 2** Proportions of new patients with head and neck cancer receiving radiology pretreatment: chest X-ray (Standard = 100% of all head and neck cancers should have a chest X-ray); CT-/MRI-scan (Standard = larynx and oral cancer 90% of T3/T4 tumours, other (ear, nose and sinus) 100%). Rates by hospital Trust. Some Trusts did not see advanced tumours in some categories, so the last three columns are blank

| Hospital Trust (code number) | % having chest X-ray pretreatment | | | % having scan pretreatment | | |
|---|---|---|---|---|---|---|
| | Larynx | Oral | Other (all) | T3/T4 Larynx | T3/T4 Oral | Other (ear, nose and sinus) |
| Regional average | 51 | 58 | 48 | 55 | 57 | 44 |
| Regional range | 0–100 | 0–91 | 0–100 | 0–100 | 0–100 | |
| 1 | 59 | 67 | 42 | 67 | 100 | 0 |
| 2 | – | – | 0 | – | | 100 |
| 3 | 25 | 64 | 35 | 0 | 44 | 33 |
| 4 | – | 0 | 100 | – | 100 | |
| 5 | – | – | 0 | – | | 0 |
| 6 | 88 | 60 | 44 | 50 | 80 | 100 |
| 7 | – | – | 0 | – | | |
| 8 | – | – | 0 | – | | |
| 9 | 100 | – | 50 | 100 | | |
| 10 | – | – | 0 | – | | |
| 11 | 50 | 0 | 67 | 0 | | |
| 12 | 100 | – | 50 | – | | |
| 13 | – | – | 0 | – | | |
| 14 | 62 | 60 | 44 | 25 | 44 | 50 |
| 15 | 15 | 25 | 30 | 100 | 0 | 100 |
| 16 | 56 | 65 | 70 | 60 | 40 | 43 |
| 17 | 10 | 80 | 27 | 50 | 80 | 0 |
| 18 | 94 | 69 | 54 | 50 | 33 | 0 |
| 19 | – | 0 | – | – | | |
| 20 | 25 | 80 | 20 | – | 100 | |
| 21 | 83 | 88 | 54 | 50 | 0 | 40 |
| 22 | 30 | 32 | 35 | 17 | 100 | 50 |
| 23 | 86 | 50 | 100 | 100 | 33 | |
| 24 | – | 0 | 0 | – | | |
| 25 | 100 | 40 | 50 | 100 | 50 | |
| 26 | 29 | 91 | 100 | 50 | 100 | |
| 27 | 50 | 50 | 27 | 100 | 100 | 0 |
| 28 | 0 | – | – | – | | |
| 29 | 17 | – | 0 | 100 | | |
| 30 | – | 67 | 33 | – | 0 | |
| Private | – | 50 | 0 | – | – | |

 © 2000 Cancer Research Campaign

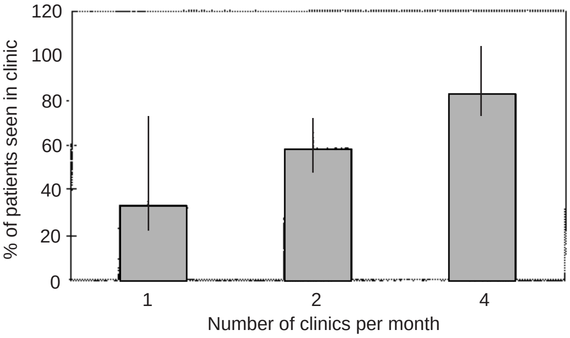

**Figure 1** Percentages of patients seen in a combined, multidisciplinary head and neck clinic pretreatment, shown by frequency of clinic. Median and range shown

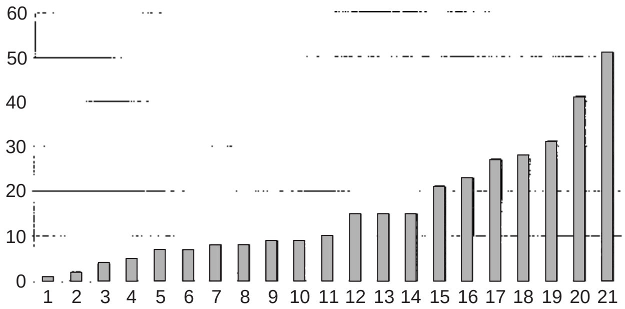

**Figure 3** Median number of cases treated per radiotherapist. There were 21 consultant radiotherapists and oncologists with a median number of new cases of 10 (range 1–51)

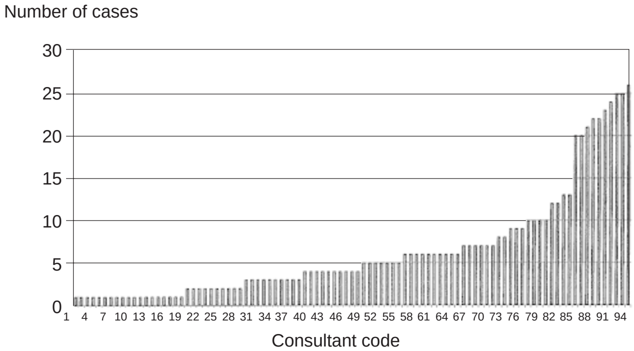

**Figure 2** Number of cases treated per surgical consultant. There were 95 surgical consultants with a median of four new patients per consultant (range 1–26)

### DISCUSSION

This study demonstrates significant differences between actual performance and established standards for the process of head and neck cancer care in the South and West of England in 1996–1997. When interpreting this data, it is important to note two points. Firstly, this audit covers new cases only and the actual activity of the hospitals may be as much as half again due to treatment of recurrent cancers. It was felt important to concentrate on new cases only since the ‘first bite at the cherry’ generally represents the best chance for effective cure or best palliation in patients with head and neck cancer. Secondly, this data was collected soon after the ‘Calman–Hine’ reforms (Calman and Hine, 1995) were announced, and in many cases, the local head and neck cancer services have undergone considerable change since the commencement of data collection in 1996. This includes the amalgamation of units in a number of hospitals. Hence, this audit should not be regarded as a measure of current practice, but rather that which was occurring at the time. Nonetheless, this represents a unique bench-marking exercise which allows us to repeat these measurements with the certain ability of measuring how far we have come.

Incidences and proportions of tumours in this study matched those predicted by retrospective cancer registry data (South and West Cancer Intelligence Unit, 1996). Completeness of data for main and secondary outcome measures was consistently over 80%.

Staging of head and neck cancers is essential for fully-informed treatment planning and prognosis, as well as being part of the necessary minimum data-set for registration and National Minimum Data-set (Wilson, 1998b; Johnson and Giles, 1999). The figure of 83% overall staging is in agreement with the 86% of clinicians who reported ‘routinely recording’ TNM stage (Edwards et al, 1997). However, although it is much higher than published rates of staging for other cancer sites (cervix, 53%) (Jackson et al, 1997; Shepherd and Quirk, 1997), lack of staging information for the remaining 17% must have severely compromised treatment-planning for this significant minority.

Only three hospital Trusts treated more than 50 new patients per annum, while 57% treated less than 20. Of these patients, approximately half received surgery, either alone or in combination. In addition, all centres had more than one treating surgeon. This is reflected in the low numbers of patients treated by most surgical consultants (median 4, 90% less than 20 per annum). While there have recently been many debates on how many patients a surgeon needs to operate on in order to maintain competence, in a complex area like head and neck cancer infrequent operating may have an adverse effect on prognosis. Local retrospective data suggest a 10% lower 5-year actuarial survival for patients whose consultant treats less than 20 patients per annum (Birchall, 1995). However, lack of staging and co-morbidity information confounds such analysis, as has been pointed out in a similar study for colorectal cancer (Kee et al, 1999). Follow-up of the present, well-characterized cohort should provide better data on this in a few years’ time.

Very few new patients (2%) in this study were referred to another hospital Trust for treatment, despite the very large differences in activity between hospital Trusts. There are many reasons for this, including financial penalties to the host Trust and, in this region, geography. Nonetheless, with increasing specialization of services, facilitated by changes in purchasing, one might expect this figure to substantially alter in the future.

Only six patients (1.1%) were treated in a private hospital, and all surgically. Of these, none received complex reconstruction. In the UK, 5–10% of all operations are performed in the private sector (BUPA figures), and the low rate for head and neck cancer probably reflects the low socio-economic grouping of these patients, as well as the need for complex multidisciplinary care which is usually only available in NHS hospitals. Inspection of these very few cases did not show any evidence to support the hypothesis that treatment selection was any different for those treated privately.

*© 2000 Cancer Research Campaign* 

**Table 3** Times between parts of the process of head and neck cancer care in the South and West of England 1996–1997 compared with established standards: larynx and oral cavity. GP = general practitioner; GDP = general dental practitioner

| Standard | Time between activities | Larynx | | | Oral cavity | | |
|---|---|---|---|---|---|---|---|
| | | Number assessed | Actual performance for region | | Number assessed | Actual performance for region | |
| | | | Median | Range | | Median | Range |
| 1 month | First symptoms to GP/GDP presentation | 159 | 3 Months | 3 days – 100 months | 144 | 2 months | 3 days – 37 months |
| 10 days | GP/GDP letter to first outpatient appointment | 143 | 21 Days | 0–395 days | 146 | 11 days | 0–78 days |
| No current standard | First outpatient appointment to biopsy | 113 | 14 Days | 0–354 days | 145 | 2 days | 0–431 days |
| 10 days | First outpatient appointment to joint head and neck clinic pretreatment | 77 | 28 days | 0–38; 9 days | 87 | 14 days | 0–698 days |
| No current standard | First outpatient appointment to first treatment date (surgery) | 37 | 26 days | 2–114 days | 103 | 29 days | 6–727 days |
| No current standard | First outpatient appointment to first treatment date (radiotherapy) | 84 | 56 days | 7–571 days | 34 | 42 days | 3–234 days |

The low proportion of patients receiving a chest X-ray, and low numbers of patients in many Trusts with advanced disease who received a CT or MRI scan are in accordance with studies of other tumour sites. Jackson reports a chest X-ray rate of 42% for patients in the South West with cervical cancer (Jackson et al, 1997), while Dickinson reports a figure of 48% for muscle-invasive bladder cancer (Dickinson et al, 1996). The availability of scanning machines and reporting expertise may be particularly limited in smaller Trusts. Nevertheless, as the established standards reflect, these investigations are fundamental to accurate staging in head and neck cancer (Houghton et al, 1998).

Times between referral and first attendance at a specialist clinic are in accordance with published figures for other cancer sites (Jackson et al, 1997; Martin et al, 1997). The longer times for laryngeal cancer than for oral cancer may reflect the vaguer nature of symptoms for many of these patients. Nevertheless, there remains an important educational message for the general public and general practitioners about the early warning signs of head and neck cancer. Overall, times for the parts of the process up to treatment were probably acceptable, and, for radiotherapy, consistent with the Royal College of Radiologists standards. However, the very long tail seen for most measurements is not. There is good biological (Wilson, 1998*a*) and clinical (Levendag et al, 1996; Dische et al, 1997) evidence that an increase in the overall time to and including treatment for head and neck cancer worsens prognosis. At an individual level, more waiting leads to more anxiety and uncertainty (Richardson, 1998).

It is generally regarded as a fundamental right of the patient with head and neck cancer to be seen and assessed before treatment planning in a multidisciplinary head and neck clinic (Tobias, 1997; Glaholm, 1997; Wilson, 1998c), and this was reflected in the standard of 95%. Thus, the overall figure of 45% is deeply disappointing. It is even lower than the figure suggested by postal survey (Edwards et al, 1997) where 56% of clinicians said they 'routinely assessed patients in joint clinics'. The present study also indicates the enormous variability in the chance of a patient being seen in such a clinic depending on where they present. A recent retrospective study from Scotland suggested that the hazard of recurrent disease, which carries a poor prognosis, is 1.9 times higher in those patients not assessed in a combined unit (Robertson et al, 1999). We agree that this pattern of care in a Western country in the 1990s is 'astonishing' (Tobias, 1997).

There was a significant relationship between the frequency of joint clinics being held and the chances of a new patient at that hospital Trust being assessed in such a clinic prior to treatment. While this seems obvious, this result has important implications. It is inconceivable that all of the hospitals in the present study have the resources (financial and manpower) to hold weekly, multidisciplinary head and neck cancer clinics, with radiotherapist and oncologist time being at the highest premium (Ryall, 1992). The inescapable message is, therefore, that only a few hospitals in each region should hold such clinics and that patients should be referred to them from other hospitals for a fully-informed, balanced and timely opinion.

Randomized trials remain the gold standard for demonstrating improvements in treatment in oncology and are regarded in some branches of oncology as one of the factors leading to improved survival figures (Stiller, 1988). However, the present study demonstrates that this is clearly not the prevailing culture in head and neck oncology. Only three patients were invited to participate in a clinical trial, and only two were actually recruited. Part of the problem is the relative rarity of these tumours and in the dilution of care among so many clinicians. Although the few trials that currently exist are not universally popular (Tobias et al, 1992), better and more relevant ones are being designed (Prof J Wilson, personal communication, 1999). Invitation to participate in a randomized controlled trial must become ingrained in the culture of head and neck clinicians, as it has in other areas of oncology (Tobias, 1997).

The lack of measurement of quality-of-life measures at diagnosis is equally disappointing, bearing in mind the recent realisation that conventional outcome measures tell only part of the story. For head and neck cancer patients, for whom treatment can be almost de-humanizing at times, it is more important than at any other cancer site that we correct this deficiency. As with clinical trials, a culture of considering the patient's quality of life before, during and after treatment will facilitate the best care for individual patients. There remains uncertainty as to which is the best of the available tools (Johnson and Giles, 1999, Rogers et al, 1998), but several of them are extremely well-validated and their use is free (Rogers et al, 1998).

*© 2000 Cancer Research Campaign*

It is possible that some of the deficiencies described in this report have been alleviated by implementation of recommendations by Trusts and Health Authorities. However, the lack of central funding to back up the reforms and the innate resistance to change of many clinicians (Sikora, 1998) makes radical improvements unlikely. In this context, the present data represents an important means of measuring local performance in this important area of oncology, facilitating effective clinical governance and gradual, incremental improvement to the care offered to this, the most unfortunate, group of cancer patients.

### ACKNOWLEDGEMENTS

The South and West RCO Tumour Panel for Head and Neck Cancer is Martin Birchall, Bristol, Chris Baughan, Southampton, Perric Crellin, Poole, Pat McLeod, Plymouth, Hugh Newman, Bristol, Mike Bridger, Plymouth, Chris Randall, Southampton, John Waldron, Bath, Nick Baker, Southampton, Tim Flood, Salisbury, Phil Guest, Bristol, Graham Zaki, Portsmouth, Peter Saxby, Exeter, John Eveson, Bristol, Julian Kabala, Bristol, Karen Forbes, Bristol, Liz Lee, Bristol, Jennifer Smith, Winchester, Kay Howe, Bristol and John Boyles, Gloucester.

The Tumour Panel gratefully acknowledge the assistance of the staff at the Cancer Intelligence Unit, Winchester, especially Sherrin Moss, for statistical analyses and coordination of the project and Regional Cancer Organisation staff for help with audit coordination and data collection, especially Veronique Poirier. The tireless secretarial assistance of Eva Hicks was invaluable. MB instigated, designed and oversaw the study, participated in quality control and wrote the paper. DB collated and analysed the data and prepared the formal report (available from the Cancer Intelligence Unit free on request). AL designed the forms, established the database and supervised the launch of the audit, as well as performing site visits. This study was part funded by a grant from the Wessex Cancer Trust. Conflict of interest: None.

### REFERENCES

Birchall M (1995) *Head and neck cancer services in Avon and Somerset: profile and proposals for development*. Report to the regional health authority. University of Bristol: Bristol

Birchall MA (ed) (1997) *Consensus standards for the process of head and neck cancer care*. South and West Regional Cancer Organisation: Bristol

Birchall MA and the South and West Expert Tumour Panel for Head and Neck Cancer (1998) Consensus standards for the process of cancer care: a modified expert panel method applied to head and neck cancer. *Br J Cancer* **77**: 1926–1931

Calman KC and Hine J (1995) A policy framework for commissioning cancer services. *A report by the Expert Advisory Group on Cancer to the Chief Medical Officers of England and Wales*. Department of Health: London

Department of Health (1996) *The protection and use of patient information*. Guidance from the Department of Health: London

Dickinson AJ, Howe K, Bedford C, Sanders T, Prentice A and Sibley GNA (1996) A retrospective study of the investigation and management of muscle-invasive bladder cancer in South West Region. *Br J Urol* **77**(1): 70–75

Dische S, Saunders M, Barrett A, Harvey A, Gibson D and Parmar M (1997) A randomised multicentre trial of CHART versus conventional radiotherapy in head and neck cancer. *Radiother Oncol* **44**: 123–136

Edwards D, Johnson NW, Cooper D and Warnakulasuriya KAAS (1997) Management of cancers of the head and neck in the United Kingdom: questionnaire survey. *BMJ* **315**: 1589

Edwards DM and Johnson NW (1999) Treatment of upper aerodigestive tract cancers in England and its effect on survival. *Br J Cancer* **81**: 323–329

Glaholm J (ed) (1997) *Quality assurance in head and neck oncology*. British Association of Head and Neck Oncologists: London

Houghton DJ, Hughes ML, Garvey C, Beasley NJ, Hamilton JW, Gerlinger I and Jones AS (1998) Role of chest CT scanning in the management of patients presenting with head and neck cancer. *Head Neck* **20**: 614–618

Jackson S, Murdoch J, Howe K, Bedford C, Sanders T and Prentice A (1997) The management of cervical carcinoma within the South West Region of England. *Br J Obstet Gynaecol* **104**: 140–144

Johnson NW and Giles A (eds) (1999) *National minimum and advisory head and neck cancer data sets*. British Association of Head and Neck Oncologists & The National Advisory Group on Screening for Oral Cancer: London

Kee F, Wilson RH, Harper C, Patterson CC, McCallion K, Houston RF, Moorehead RJ, Slaon JM and Rowlands BJ (1999) Influence of hospital and clinician workload on survival from colorectal cancer: cohort study. *BMJ* **318**: 1381–1386

Levendag PC, Nowak PJ, van der Sangen MJ, Jansen PP, Eijkenboom WM, Planting AS, Meeuwis CA and van Putten WL (1996) Local tumor control in radiation therapy of cancers in the head and neck. *Am J Clin Oncol* **19**: 469–477

Martin IG, Young S, Sue-Ling H and Johnston D (1997) Delays in the diagnosis of oesophagogastric cancer: a consecutive case series. *BMJ* **314**: 467–470

Richardson A (1998) *Finding their voices. The views of persons who have had a laryngectomy on the process of head and neck cancer care*. South and West Regional Cancer Organisation: Bristol

Robertson AG, Robertson C, Soutar DS, Burns H, Hole D and McCarron P (1999) *Treatment of oral cancer: the need for defined protocols and specialised centres*. Presented at the Annual General Meeting of the British Association of Head and Neck Oncologists: London

Rogers SN, Lowe D, Brown JS and Vaughan ED (1998) A comparison between the University of Washington Head and Neck Disease-Specific measure and the Medical Short Form 36, EORTC QLQ-C33 and EORTC Head and Neck 35. *Oral Oncol* **34**: 361–372

Ryall RD (1992) Medical manpower and workload in clinical oncology in the United Kingdom. *Clin Oncol* **4**: 69–70

Shepherd NA and Quirke PJ (1997) Colorectal cancer reporting: are we failing the patient? *Clin Pathol* **50**: 266–267

Sikora K (1998) Cancer reforms lag behind. *Hospital Doctor* September: 42–43

South and West Cancer Intelligence Unit (1996) Factsheet No. 12: *Head and neck cancer in the South and West*. South and West Cancer Intelligence Unit: Winchester

Stiller CA (1988) Centralisation of treatment and survival rates for cancer. *Arch Dis Child* **63**: 23–30

Tobias JS, Selby PJ, Gupta NK, Rhys-Evans PH, Peto J and Houghton J (1992) *UKHAN I*. UKCCR Head and Neck Collaborative Group: London

Tobias JS (1997) Management of head and neck cancer in Britain. Plenty of room for improvement. *BMJ* **315**: 1556

Union Internationale Contre le Cancer (1997) *TNM atlas*, 4th edn. Springer: Berlin pp 5–63

Wilson GD (1998*a*) Tpot and head and neck cancer: where are we now? (1998) *Anticancer Res* **18**: 4801–4805

Wilson J (ed) (1998*b*) *Effective Head and Neck Cancer Management*. British Association of Otorhinolaryngologists Head and Neck Surgeons: London: pp 21–22

Wilson J (ed) (1998*c*) *Effective Head and Neck Cancer Management*. British Association of Otorhinolaryngologists Head and Neck Surgeons: London: pp 30–34

Wilson J (ed) (2000) *Effective Head and Neck Cancer Management*, 2nd Edn. British Association of Otorhinolaryngologists Head and Neck Surgeons: London

© 2000 Cancer Research Campaign
